# Ferroptosis Activation Contributes to the Formation of Skin Lesions in Psoriasis Vulgaris

**DOI:** 10.3390/antiox12020310

**Published:** 2023-01-29

**Authors:** Siying Li, Xin Luo, Suhan Zhang, Yuwen Su, Min Deng, Yanshan Zhu, Peng Zhang, Ruifang Wu, Ming Zhao

**Affiliations:** 1 Hunan Key Laboratory of Medical Epigenomics, Department of Dermatology, The Second Xiangya Hospital of Central South University, Changsha 410011, China; 2 Research Unit of Key Technologies of Diagnosis and Treatment for Immune-Related Skin Diseases, Chinese Academy of Medical Sciences, Changsha 410011, China; 3 Clinical Medical Research Center of Major Skin Diseases and Skin Health of Hunan Province, Changsha 410011, China

**Keywords:** ferroptosis, psoriasis, keratinocyte, lipid peroxidation, iron overload, Cyb561d2

## Abstract

(1) Background: Ferroptosis is a newly coined form of programmed cell death marked by lethal accumulation of lipid peroxidation and ferrous iron overload. A few studies on the specific mechanism of ferroptosis in the genesis and development of psoriasis are available. (2) Methods: Levels of lipid reactive oxygen species (ROS) and ferrous iron were measured by flow cytometry. Ultrastructure analysis was performed by transmission electron microscopy. Imiquimod-induced psoriasis-like mice were treated with a ferroptosis inducer. The expressions of mRNA of genes were measured by qRT-PCR. HaCaT cells were used to explore the function of Cyb561d2. (3) Results: In this work, we observed that levels of lipid ROS and ferrous iron in the epidermis of psoriasis vulgaris (PV) patients were increased. The existence of ferroptosis activation in the epidermis of individuals with PV was confirmed by transmission electron microscope both in patients with PV and psoriasis-like mice models. Intradermal injection of the ferroptosis inducer RSL3 in psoriasis-like mice significantly promoted and aggravated the development of psoriasis-like dermatitis, and the level of serum transferrin was also increased in PV samples. Moreover, abnormal expression of some genes related to iron metabolism was also proved in the epidermis of PV cases, among which Cyb561d2 was shown to promote ferrous iron overload and lipid peroxidation accumulation in HaCaT cells. (4) Conclusions: In summary, our study suggested that ferroptosis activation owing to iron overload may be a novel mechanism underlying the formation of skin lesions in individuals with PV.

## 1. Introduction

Psoriasis is a chronic and systemic skin disease characterized by erythematous plaques covered with silvery scales [1,2]. Dysfunction of epidermal keratinocytes including excessive hyperproliferation and aberrant differentiation play crucial roles in psoriasis pathogenesis [3]. Keratinocytes are the major cells in the epidermal barrier, which defends against invasive pathogens and skin injury [4]. Abnormal activation of keratinocytes can release abundant vascular endothelial growth factor (VEGF) [5]. The VEGF can enhance the density, permeability, and dilatation of dermal capillaries in psoriasis lesions, leading to a hyperproliferative epidermis [6,7]. Moreover, activated keratinocytes can release massive chemokines and indirectly recruit immune cells. In turn, immune cells produce a variety of proinflammatory cytokines, such as IL-17A, IL-17F, IL-23, TNF-α, and IFN-γ, and those act on keratinocytes [8,9,10]. These changes result in an inflammatory cascade in the psoriatic lesions and promote the development of psoriasis. Thus, epidermal keratinocytes are essential in the pathogenesis of psoriasis, both as inflammatory triggers and immune responders.

Ferroptosis is a form of programmed cell death newly defined in 2012, and characteristic biochemical changes are the superfluous accumulation of lipid peroxidation and ferrous iron overload [11,12,13]. Thus far, the mechanism of ferroptosis remains unclear but the iron content and status, glutathione, free oxygen radicals, ROS, and lipid peroxides are closely related to ferroptosis. The antioxidant system is destroyed in ferroptotic cells, which is reflected in the abnormal expression of numerous genes related to lipid oxidation metabolism and the enhancement of products of lipid peroxidation, including lipid ROS and malondialdehyde [14,15]. A Fenton reaction occasioned by intracellular ferrous iron overload can directly generate extreme ROS, thereby increasing the sensitivity of ferroptosis [11]. Recent evidence indicated that redox imbalance towards a peroxidative state played a crucial role in the pathophysiology of psoriasis [16,17]. Previous studies proved that the levels of MDA in plasma and skin lesions of psoriasis patients were higher than healthy controls, especially in the acute stage or severe conditions [18,19,20]. Specifically, the latest study confirmed that the inhibition of ferroptosis by ferrostatin-1 suppresses psoriasis lesions in mice [21]. However, the mechanism of ferroptosis in the pathogenesis of psoriasis remains poorly understood.

In this work, we demonstrated that the levels of lipid ROS and ferrous iron were increased in the epidermis of PV patients compared to healthy controls. Cells in the epidermis of PV patients showed ferroptosis-like abnormalities of mitochondria under transmission electron microscope. Moreover, enhancing the sensitivity of ferroptosis by intradermal injection of a ferroptosis inducer can promote and aggravate dermatitis in an imiquimod (IMQ)-induced psoriasis-like mouse model. Furthermore, Cyb561d2 showed abnormally high expression in the psoriatic lesion, and overexpression of Cyb561d2 in HaCaT cells can induce the ferrous iron overload and lipid peroxidation. Our findings verified a novel insight into programmed cell death in PV cases and proposed that ferroptosis activation in the epidermis of PV patients may originally be facilitated by ferrous iron overload through Cyb561d2 upregulation.

## 2. Materials and Methods

### 2.1. Human Subjects

This study obtained permission from the Ethics Committee of the Second Xiangya Hospital of Central South University (Changsha, Hunan, China). All volunteers signed the written informed consent. All patients with PV who participated in the study were diagnosed with PV by histopathologic biopsy in the pathological center of the Second Xiangya Hospital of Central South University. Psoriatic lesions were donated by 37 adult patients, and healthy skin samples were collected from 46 healthy donors. Peripheral blood was obtained from 188 patients and 124 healthy controls. 

### 2.2. Animal Studies

#### 2.2.1. Psoriasis-like Mouse Model Induced by IMQ

Female BALB/c mice of the same age were supplied by Slack company (Shanghai, China) and were used to perform the psoriasis-like mouse model by IMQ. Every day, 62.5 mg of 5% IMQ cream (Med-shine Pharmaceutical, Sichuan, China) was applied to the shaved dorsal skin for 6 consecutive days. The same dose of vehicle cream was treated to control mice.

#### 2.2.2. RSL3 Induction

The mice were intradermally injected with 2 mg/kg of RSL3 dissolved in 50 μL saline or 50 μL saline alone daily after application of IMQ for 6 consecutive days. On day 4 and day 7, skin lesions were analyzed by histopathology, transmission electron microscopy, and immunofluorescence analysis. 

### 2.3. Epidermis Collection and Digestion

Fresh skin samples were cut off the hypodermis with eye scissors and then washed by HBSS. The skin samples were cut into small pieces and they were incubated in 5 mg/mL dispase II (Roche) at 4 °C for 1 h. Skin samples were transferred to a plate containing 2–3 mL HBSS and the epidermis was gently scraped off from the dermis. The epidermis was collected in a new tube and digested with trypsin. The suspension was passed through 40 μm mesh after termination of digestion and epidermal keratinocytes were collected.

### 2.4. qRT-PCR

Cells and skin samples were used to extract total RNA with TRIzol reagent (Takara, Kusatsu, Japan). NanoDrop spectrophotometer (ND-2000, Thermo Fisher Scientific, Waltham, MA, USA) detected the quality of RNA. cDNA was synthesized by reverse-transcribed mRNA with the PrimeScript RT reagent kit with gDNA Eraser (Takara, Japan), and the quality ceiling of RNA was 1 μg. A LightCycler 96 (Roche, Rotkreuz, Switzerland) thermocycler was used to carry out qPCR with the SYBR Premix Ex Taq II (Takara, Japan). The relative expression of genes in the skin samples were figured with the 2-△Ct method, and the relative expression of Cyb561d2 in HaCaT cells was analyzed with the 2-△△Ct method. The sequences of all primers used in this study are summarized in Figure S1. 

### 2.5. Western Blotting

Skin samples were lysed in radio immunoprecipitation assay buffer supplemented with protease and phosphatase inhibitor (Beyotime). Proteins were quantified by the Bradford assay (HyClone-Pierce). Rabbit anti-Cyb561d2 Ab (0.2 μg/mL; Novus biology; catalog NBP1–89415) and Rabbit anti-GAPDH Ab (1:2000; Abcam; ab9485) were used. Data were analyzed using a GE-ImageQuant LAS 4000 mini (GE Healthcare). Quantification of Cyb561d2 was normalized to GAPDH by densitometry. Images were cropped for presentation.

### 2.6. Analysis of Lipid Peroxidation and Intracellular Ferrous Iron

For lipid peroxidation analysis, cells were stained with a probe of lipid ROS with a concentration of 10 μmol/L, which we purchased from Thermo Fisher Scientific (named BODIPY-581/591 C11), in complete culture medium in a 37 °C incubator for at least 30 min. Next, cells were washed by glacial PBS twice at least, then immediately analyzed by flow cytometry. Relative value of lipid peroxidation levels was calculated by the ratio of the mean fluorescence intensity (MFI) of FITC channel to MFI of PE channel [22]. For intracellular ferrous iron detection, the cell pellet was washed and resuspended with HBSS or serum-free medium, and then stained with 1 μmol/L FerroOrange (DOJINDO, Tokyo, Japan) in a 37 °C incubator for 30 min followed by flow cytometry analysis. The MFI of PE channel was recorded [23]. 

### 2.7. Histological Analysis and Immunofluorescence

Mouse skin tissues were fixed in formalin and embedded in paraffin. 6-μm-thick sections were prepared for the following staining. For H&E analysis, ten-minute hematoxylin staining followed by two-minute eosin staining was performed. The skin sections were stained with hematoxylin for 10 min and then stained with eosin for 2 min. We scored acanthosis to assess the degree of epidermal hyperplasia. The calculation of acanthosis was based on the ratio of the pixel size of the epidermal area to that of the entire image. For immunofluorescence, deparaffinization was performed followed by heat-induced antigen retrieval. Rabbit anti-K i67 polyclonal antibody (1:200, #12202, Cell Signaling Technology) was used to stain. Next, the secondary antibody and Opal 540 Fluorophores were orderly incubated for 20 min. Finally, the quantitative Pathology Imaging System (PerkinElmer, Waltham, MA, USA) was used to capture the image. 

### 2.8. Cell Culture

HaCaT cells were obtained from National Collection of Authenticated Cell Cultures and were maintained in DMEM medium (Gibco) with 15% fetal bovine serum (FBS, Gibco) at 37 °C and 5% in a CO_2_ incubator.

### 2.9. siRNA or Plasmid Transfection of HaCaT Cells

For siRNA or plasmid transfections, HaCaT cells were transfected with siRNA or plasmid using Lipofectamine (Thermo Fisher Scientific). Generally, HaCaT cells were cultured in a dish and treated with 20 μM siRNA or 4 μg plasmids. After incubation for 48 or 72 h, RT-qPCR verified the transfection effect. The target sequence of Cyb561d2 siRNA was GGTGAGCAATGCCTACCTA.

### 2.10. Statistical Analysis

More than three independent experiments were implemented to obtain all data. GraphPad Prism 8.0 software was utilized for statistical analysis and a *p* value < 0.05 indicated statistical significance. Normality of distribution and similar variance between groups was assessed for data. Between the two groups, comparisons were evaluated by two-tailed unpaired Student’s t-test. If there was no normal distribution or the variance was unequal among the two groups, we used Mann–Whitney *U* test was used to analyze the data. For correlation analysis, Spearman’s test was used.

## 3. Results

### 3.1. Activation of Ferroptosis Existing in the Epidermis of PV Patients

**Figure 1:**
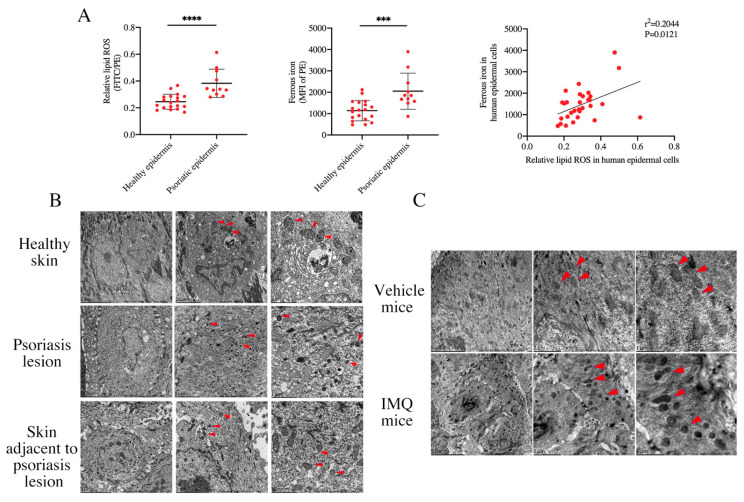
Ferroptosis is activated in the epidermis of psoriasis vulgaris samples. (**A**) Levels of lipid peroxidation and ferrous iron (MFI of PE channel) in epidermis samples derived from normal controls (*n* = 19) and psoriatic skin lesions (*n* = 11), the relationship between lipid peroxidation and ferrous iron. (**B**) Representative TEM images of normal control skin, psoriatic skin lesion, and its adjacent skin. Red triangles indicate mitochondria. Scale bars: 5 μm (left), 2 μm (middle), and 1 μm (right). (**C**) Representative TEM images of shaved dorsal skin from IMQ-treated mice and vehicle-treated mice. Red triangles indicate ferroptosis-related mitochondria. Scale bars: 5 μm (left), 2 μm (middle), and 1 μm (right). Data represent the mean ± SEM: *** *p* < 0.001, **** *p* < 0.0001. Two-tailed Mann–Whitney U test was used.

To prove the existence of ferroptosis in PV patients, we investigated the classical ferroptosis-related markers. Firstly, we measured the level of lipid ROS and ferrous iron in the epidermal cells in PV patients. As expected, our results showed that compared to healthy controls, the level of intracellular lipid ROS and ferrous iron in epidermis of PV patients were increased, and there was a significant positive correlation (Figure 1A). Ferroptosis was distinguished from other types of programmed cell death such as pyroptosis, necrosis, and autophagy mainly, reflected in morphological changes. Ferroptotic cells manifested as the volume of mitochondria reduced, mitochondria crista decreased or disappeared, the membrane density of mitochondria increased, or the outer membrane of mitochondria ruptured, shown through transmission electron microscopy [24,25,26]. Transmission electron microscopy ultrastructure analysis presented that the epidermis cells of PV patients exhibited more ferroptotic ultrastructure than those with healthy epidermis (Figure 1B), which means that ferroptosis was activated in the epidermis of the psoriatic lesion. More interestingly, mitochondria in the normal-appearance skin adjacent to the psoriasis existed with increased density of partial membrane. Similar to the human subjects, the skin lesion from IMQ-induced psoriasis-like mice also showed characteristically morphological features of ferroptosis (Figure 1C). In summary, these results demonstrated that ferroptosis was activated in the epidermis of psoriatic lesions. 

### 3.2. Ferroptosis Induced by RSL3 Promotes the Formation of Skin Lesions in IMQ-Induced Psoriasis-like Mice

**Figure 2:**
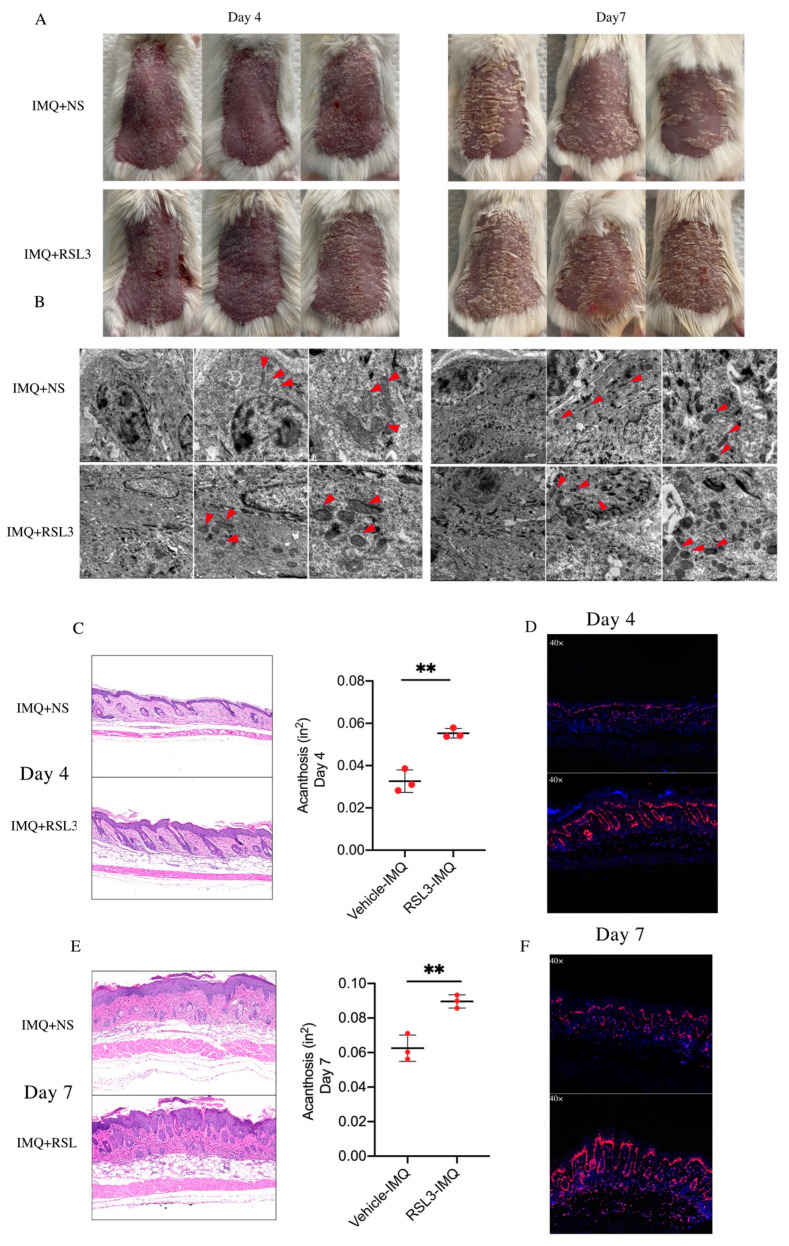
IMQ-induced psoriasis-like mice stimulated by ferroptosis inducer RSL3. (**A**) Phenotypic presentation of lesional skin from mice injected with RSL3 and saline on day 4 and day 7. (**B**) Representative TEM images of epidermis tissues derived from mice injected with RSL3 and saline on day 4 and day 7 (red triangles indicate mitochondria). (**C**) Representative H&E staining image and acanthosis assessment of lesions from mice injected with RSL3 and saline on day 4. Scale bars: 100 μm. Acanthosis of mice treated with NS (*n* = 3) or RSL3 (*n* = 3). (**D**) Representative immunohistochemical analysis of Ki-67+ cells in skin samples on day 4. (**E**) Representative H&E staining image and acanthosis assessment of lesion from mice injected with RSL3 and saline on day 7. Acanthosis of mice treated with NS (*n* = 3) or RSL3 (*n* = 3). Scale bars: 100 μm. (**F**) Representative immunohistochemical analysis of Ki-67+ cells in skin samples on day 7. Data represent the mean ± SEM, ** *p* < 0.01. Two-tailed Mann—Whitney U test was used.

Our previous findings indicated that ferroptosis was activated in psoriatic lesions. RSL3 (AS-selective lethal), as a classical ferroptosis inducer, can effectively enhance ferroptosis sensitivity of cells through irreversibly inactivating glutathione peroxidase 4 (GPX4). To further explore the exact role of ferroptosis in the formation of psoriatic lesions, we intradermally injected RSL3 or normal saline (NS) daily in the dorsal skin of IMQ-induced psoriasis-like mice. Compared to the NS group, the RSL3 group showed accelerated pathological progression of psoriasis-like lesions and delayed skin injury repair (Figure 2A). During the process of the experiment, we discovered that the characteristic ferroptotic ultrastructure was significantly induced on day 4 and day 7 in the RSL3 group compared to the NS group, along with more severe pathological and clinical changes (Figure 2B). The epidermal thickness on the dorsal skin of the RSL3 group was enhanced both on day 4 and day 7 (Figure 2C,E). Interestingly, compared with the NS group, the percentage of Ki67-positive cells on the psoriatic lesion was higher in the RSL3 group on day 4, but there was no difference on day 7 (Figure 2D,F). Together, these findings indicate that the induction of ferroptosis in psoriasis-like mice promoted the development of psoriatic lesions and increased disease severity.

### 3.3. Ferrous Iron Overload Contributes to Ferroptosis Activation of Psoriatic Epidermis

**Figure 3:**
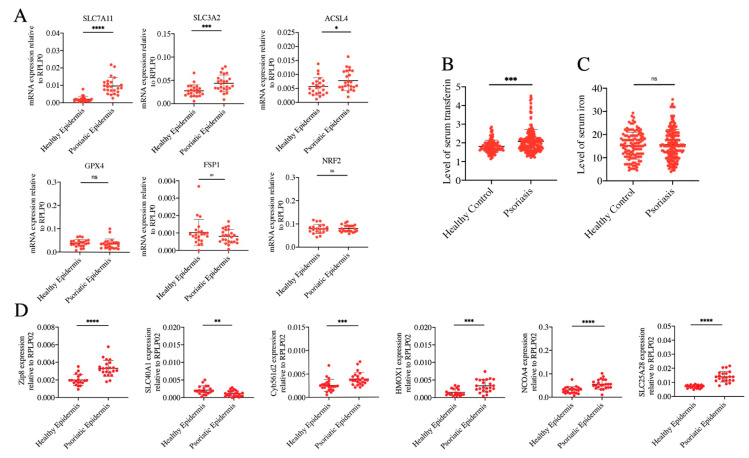
Stage of lipid peroxidation and iron metabolism in the epidermis of PV patients. (**A**) Lipid-peroxidation-related genes in the psoriatic epidermis (*n* = 23) and normal controls (*n* = 24). (**B**,**C**) Levels of serum iron and serum transferrin in psoriasis patients (*n* = 177) and normal controls (*n* = 115). (**D**) Iron-metabolism-related genes in the psoriatic epidermis (*n* = 23) and normal controls (*n* = 24). Data represent the mean ± SEM: ^ns^ *p* > 0.05, * *p* < 0.05, ** *p* < 0.01, *** *p* < 0.001, and **** *p* < 0.0001. Two-tailed Mann–Whitney U test was used.

Lethal lipid peroxidation and excessive iron accumulation are the hallmarks of ferroptosis. Thus, abnormal expression or dysfunction of genes which regulate lipid peroxidation or maintain iron homeostasis contributes to ferroptosis activation to cells. To elucidate the mechanism that was responsible for the induction of ferroptosis in the development of psoriasis, we detected the expression of classically ferroptotic genes related to lipid peroxidation signaling pathways involving GPX4, nuclear factor erythroid 2-like 2 (NRF2), acyl–coenzyme A synthetase long-chain family member 4 (ACSL4), apoptosis-inducing factor mitochondria-associated 2 (AIFM2/FSP1), and system xc (SLC7A11/SLC3A2). The mRNA expressions of SLC7A11, SLC3A2, and ACSL4 were upregulated in the epidermis of PV, but there was no difference in the expression of GPX4, FSP1, and NRF2 between the epidermis of PV and normal controls (Figure 3A). These results suggested that unregulated expression of genes related to lipid peroxidation may not be the initial cause of enhancement of lipid ROS and ferroptosis in the epidermis of PV. 

Ferrous iron overload can cause ferroptosis. In theory, intervention at any step of iron metabolism can induce ferrous iron overload, including absorption, storage, utilization, and flux. Serum transferrin in human blood can transport iron into tissues and organs. We found that compared to healthy controls, serum transferrin was significantly increased in PV cases, although the contents of serum iron were unchanged (Figure 3B,C). In the epidermis of PV cases, expression of genes related to iron import, including solute carrier family 39 member 8 (Zip 8) and solute carrier family 25 member 28 (SLC25A28) were increased, while the expression of SLC40A1 which exports iron was decreased. Moreover, genes that can facilitate ferrous iron generation were upregulated, involving in ferrireductases (cytochrome b561 family member D2, Cyb561d2), heme oxygenase 1(HMOX1), and nuclear receptor coactivator 4 (NCOA4) (Figure 3D). These data were consistent with upregulation of lipid ROS and ferrous iron in the epidermis of PV samples, which suggested that ferrous iron overload might be the initial cause of ferroptosis activation of psoriatic epidermis.

### 3.4. Overexpression of Cyb561d2 Is Involved in Ferroptosis in HaCaT Cells

**Figure 4:**
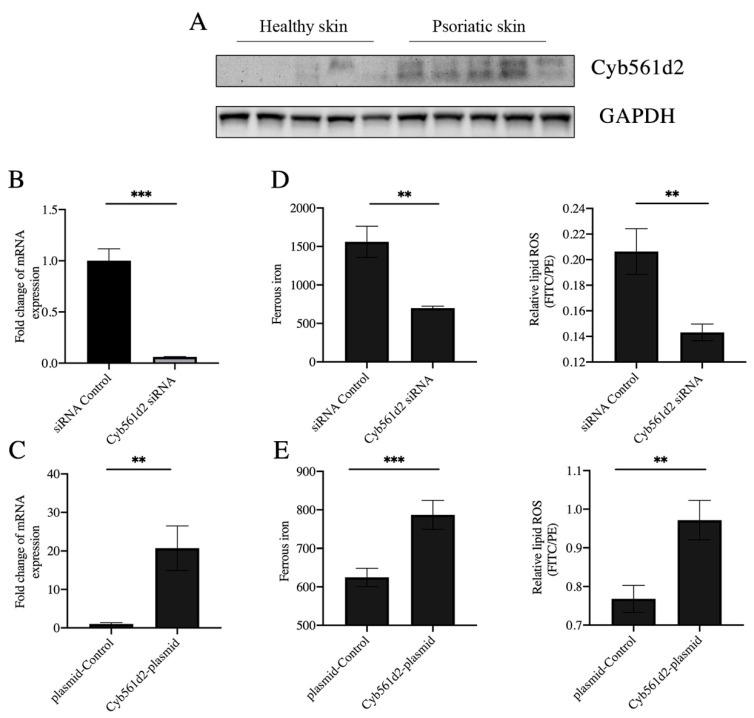
Level of lipid ROS and iron in HaCaT cell. (**A**) Protein level of Cyb561d2 measured by Western blot in psoriatic skin lesions (*n* = 5) and normal controls (*n* = 5). (**B**) Test for knockdown efficiency of Cyb561d2 siRNA in HaCaT cells. (**C**) Test for overexpression efficiency of Cyb561d2 plasmid in HaCaT cells. (**D**) Levels of intracellular ferrous iron and levels of lipid peroxidation in HaCaT cells transfected with Cyb561d2 siRNA (*n* = 3) and control siRNA (*n* = 3). (**E**) Levels of intracellular ferrous iron and levels of lipid peroxidation in HaCaT cells transfected with DNA plasmid (*n* = 3) and control plasmid (*n* = 3). Data represent the mean ± SEM: ** *p* < 0.01, and *** *p* < 0.001. Two-tailed Mann–Whitney U test was used.

A recent study reported that CYB5R1 can induce ferroptosis by promoting the iron-dependent Fenton reaction [27]. However, there was no significant difference in expression of CYB5R1 between psoriasis epidermis samples and healthy controls (Figure S2B). Cyb561d2, a homologue of CYB5R1 and potent antioxidant, is a member of the cytochrome b561 family and can catalyze ferric iron reduction [28,29,30]. Thus, the upregulation of Cyb561d2 is effective in the generation of ferrous iron. However, there was no report on the role of Cyb561d2 in psoriasis or ferroptosis. A recent study reported that CYB5R1, a homologue of Cyb561d2, can induce ferroptosis by promoting the iron-dependent Fenton reaction [31]. We detected the level of Cyb561d2 by Western blot, and the results showed that the protein expression of Cyb561d2 was increased in the epidermis of psoriatic lesions (Figure 4A). Based on the findings of the significant upregulation of Cyb561d2 in the epidermis from the psoriatic lesion, we transfected HaCaT cells, an immortalized human keratinocyte line, with the Cyb561d2 siRNA or Cyb561d2 overexpression plasmid. The results demonstrated that the expression of Cyb561d2 was suppressed by Cyb561d2 siRNA and enhanced by the Cyb561d2 overexpression plasmid (Figure 4B,C). Moreover, the levels of ferrous iron were reduced by Cyb561d2 knockdown with siRNA transfection, along with decreased content of lipid ROS (Figure 4D). Consistent with this, Cyb561d2 overexpression promoted the generation of ferrous iron in HaCaT cells and led to induced lipid peroxidation (Figure 4E). Taken together, these results indicated that Cyb561d2 mediated the process of lipid ROS throughout the generation of ferrous iron.

## 4. Discussion

Studies on the pathogenesis of psoriasis have shown that keratinocyte homeostasis is critical in the formation of psoriasis lesions. Various types of programmed cell death regulated the proliferation and differentiation of keratinocytes in psoriasis. Ferroptosis is a novel form of programmed cell death, which is driven by the accumulation of lipid peroxidation and ferrous iron overload. Implications of ferroptosis in several immune-mediated diseases including ulcerative colitis, Crohn’s disease, and systemic lupus erythematosus have been proven [31,32,33]. In addition, extreme ROS plays a crucial role in the progression of psoriasis [34,35,36]. However, the precise mechanism underlining the effect of ferroptosis on psoriasis remains poorly understood. Here, we demonstrated that ferroptosis was activated in the epidermis both in psoriasis patients and psoriasis-like mouse models, reflected in excessively accumulated lipid ROS and ferrous iron and the obvious ferroptotic ultrastructure. More interestingly, the normal-appearance skin adjacent to psoriasis lesions showed a partially ferroptotic ultrastructure. This suggested that metabolites from the psoriatic lesion area might progressively attack the surrounding normal keratinocytes, further contributing to the development of psoriatic lesions. For ferroptosis in psoriasis cases, the products included in lipid ROS and ferrous iron gradually spread to normal skin and promoted the formation of psoriasis lesions. Enhanced ferroptosis induced by ferroptosis inducers promoted the development of psoriatic lesions and increased disease severity in psoriasis-like mouse models. Moreover, we preliminarily found that ferrous iron overload rather than lipid peroxidation might be the initial induction of ferroptosis in the psoriatic epidermis. As a ferrireductase protein, the expression of Cyb561d2 was upregulated in the epidermis of PV cases. Indeed, Cyb561d2 overexpression induced the levels of ferrous iron, accompanied by an increase in lipid ROS. Thus, our data indicated that iron overload by Cyb561d2 overexpression might contribute to the activation of ferroptosis and formation of psoriatic lesions. 

Lipid peroxidation is a necessary process in ferroptosis. In recent years, studies have verified that ROS contribute to the development of psoriasis, and antioxidant therapy has been effective in relieving psoriatic lesions [14,37]. In this study, ferroptosis with higher levels of lipid ROS has been proven exist in the epidermis of individuals with psoriasis. Therefore, we hypothesized that genes related to lipid peroxidation might be a primary trigger in the ferroptosis of psoriasis. GPX4 can reduce the cytotoxic lipid peroxide to the nontoxic corresponding lipid alcohol and then clean the lipid peroxidation. Initial studies have shown that dysfunction of GPX4 primarily drove ferroptosis through depriving its ability to scavenge lipid peroxidation products [12]. However, there were no differences in the mRNA and protein expression of GPX4 found between the psoriatic epidermis and healthy controls (Figure S2B). System Xc, one of the amino acid transporters, transports cystine into the cell and synthesizes reduced synthetic glutathione, which promotes the removal of lipid peroxidation products by GPX4 and confronted ferroptosis [38]. Unexpectedly, SLC7A11 was upregulated in the psoriatic epidermis. These results suggested that the accumulation of lipid ROS in the psoriatic epidermis may not be caused by abnormal expression of genes related to lipid peroxidation.

Another hallmark of ferroptosis is ferrous iron overload. Ferrous iron, as a redox-active metal, can catalyze the generation of ROS with high reactivity by the Fenton reaction and lead to oxidative damage. Interestingly, the Fenton reaction is driven by ferrous iron overload, which then can induce lipid peroxidation and ultimately drive ferroptosis. In this study, we found that the level of intracellular ferrous iron was increased in the psoriatic epidermis and positively correlated with the level of lipid ROS. At the systemic level, transferrin can bind with iron in the circulation and deliver it into tissues and organs throughout the body, thus transferrin abundance partially reflects iron availability. Under normal physiological conditions, transferrin transports iron into cells upon receiving signal of excess iron in the peripheral circulation. However, our results presented that there were no differences in serum iron, but the serum level of transferrin was significantly elevated in psoriasis cases, which meant that iron metabolism in psoriasis was abnormal. Moreover, we detected the expression of genes related to iron metabolism in the epidermis of individuals with PV and found that many of them were differentially expressed, compared with healthy controls. In the epidermis of PV cases, Zip8, which transports ferrous iron into cells, was upregulated, but SLC40A1/FPN1, which is the only known iron exporter, was downregulated. Such regulation would lead to ferrous iron overload in the epidermis of PV patients. Meanwhile, genes that can enhance the abundance of intracellular ferrous iron by directly releasing ferrous iron from ferritin or reducing ferric iron to ferrous iron, including in Cyb561d2, HMOX1 and NCOA4, were upregulated in the epidermis of individuals with PV. Moreover, mitoferrin-2, also known as SLC25A28, which can transport iron into mitochondrion and increase mitochondrial iron accumulation, was also upregulated. We speculated that ferrous ions in cytoplasm and mitochondria of psoriatic epidermal keratinocytes were elevated, which eventually led to ferroptosis in the psoriatic epidermis. Furthermore, we hypothesized that ferrous iron overload in the epidermis might be the original cause of ferroptosis activation of PV patients. 

A novel finding in our study was the potential function of Cyb561d2 in psoriasis. A previous study reported that Cyb561d2 can inhibit the genesis and development of lung cancer by its inhibitory effects on cell growth [39]. At that time, researchers defined the inhibitory effects as caspase-independent apoptosis and autophagy without the concept of ferroptosis. Interestingly, Cyb561d2 knockdown reduced the content of ferrous iron along with decreased lipid ROS. Similarly, Cyb561d2 overexpression induced the content of ferrous iron along with increased lipid ROS in HaCaT cells. Psoriasis is a chronic, recurrent, and persistent skin disease. Perhaps, long-term accumulation of constant ferrous iron overload that was mediated by Cyb561d2 upregulation leads to lipid peroxidation, and then activates ferroptosis in keratinocytes. Thus, we speculated that Cyb561d2 might regulate ferrous iron generation to affect lipid peroxidation in ferroptosis activation in psoriasis. 

There were some limitations in our study. Firstly, we can further observe whether the application of iron chelators can reduce the formation of psoriasis lesions in the psoriasis-like mouse model. Secondly, we only showed three repeated mice models, even though we verified multiple times that RSL3 can accelerate and aggravate the formation of psoriatic lesions. Thirdly, we will construct Cyb561d2 keratinocyte-specific knockout mice to further explore the role of Cyb561d2 in the formation of psoriatic lesions. Moreover, IL23A-IL17A axis activation is necessary for the development of psoriasis. In the future, the association between Cyb561d2 upregulation and IL23A-IL17A axis activation must be explored.

## 5. Conclusions

In conclusion, this study confirmed the existence of activated ferroptosis in the epidermis of psoriasis vulgaris samples, and the induction of ferroptosis can promote the development of lesions in psoriasis-like mice. Furthermore, this study indicated that aberrant iron metabolism might be the original cause of ferroptosis activation in psoriatic keratinocytes and provided novel clues to elucidate the pathogenesis of psoriasis vulgaris. The underlying molecular mechanisms need to be investigated in future research.

## Data Availability

The data used to support the findings of this study are available from the corresponding authors upon request.

## Supplementary Materials

The following supporting information can be downloaded at: https://www.mdpi.com/article/10.3390/antiox12020310/s1.
