# Supplementary material for: Ferroptosis Activation Contributes to the Formation of Skin Lesions in Psoriasis Vulgaris
Source: Antioxidants (Basel). 2023 Jan 29;12(2):310. doi: 10.3390/antiox12020310 (PMC9952139; doi:10.3390/antiox12020310)
Supplement: Supplementary file 1 [file antioxidants-12-00310-s001.zip › antioxidants-2109138-supplementary/figure S1.pdf]

| Gene Symbol | Primer Sequence |
|---|---|
| RPLP02-FP | TGGTCATCCAGCAGGTGTTCGA |
| RPLP02-RP | CAGACACTGGCAACATTGCGG |
| FSP1-FP | AGGGCCACAGCAACGTCTA |
| FSP1-RP | CCATGGACAGGAGGAACGTC |
| GPX4-FP | GCGGGCTACAACGTCAAATT |
| GPX4-RP | CAGCCGTTCTTGTCGATGAG |
| SLC7A11-FP | CAGCTGGTAGAGGAGTGTGC |
| SLC7A11-RP | CTCCTGCTTTGGCTCCATGA |
| SLC3A2-FP | CTGGTGCCGTGGTCATAATC |
| SLC3A2-RP | GCTCAGGTAATCGAGACGCC |
| ACSL4-FP | CCCGCTATCTCCTCAGACAC |
| ACSL4-RP | ATAACCGCCTTCTTGCCAGT |
| NRF2-FP | CTCCACAGAAGACCCCAACC |
| NRF2-RP | AAGTAGCAGGTGAGGGCATG |
| DMT1-FP | TCATGCTGGCCTCTTTCCTA |
| DMT1-RP | AGGTTCCTGTCATGGTGGAG |
| Transferrin Receptor-FP | ATGTTGTAGTTGGGGCCCAG |
| Transferrin Receptor-RP | ATTCAGTGGCACCAACCGAT |
| Zip8-FP | TGCCTGGATGATAACGCTCT |
| Zip8-RP | CTTGTCGAGTGCTCATCCCT |
| Zip14-FP | TATGCCTCTGAGTCGCTTCC |
| Zip14-RP | AGTAGCAAGCACTCTGGGAA |
| SLC40A1-FP | CTACTTGGGGAGATCGGATGT |
| SLC40A1-RP | CTGGGCCACTTTAAGTCTAGC |
| SLC25A37-FP | CTCCACGATGCGGTAATGAA |
| SLC25A37-RP | GTGGTGTAGCTCCGGTAGAA |
| SLC25A28-FP | CAGCTGACCATGAACGTTCC |
| SLC25A28-RP | CTGGGTGTTGAGCAGTGTTT |
| CYB561D2-FP | CTCGGCCTTGTCATCCTCC |
| CYB561D2-RP | CCCACCAGCCCAGAAGTAG |
| HMOX1-FP | GGTCATCCCCTACACACCAG |
| HMOX1-RP | CAGACAGGTCACCCAGGTAG |
| NCOA4-FP | GAGGTGTAGTGATGCACGGAG |
| NCOA4-RP | GACGGCTTATGCAACTGTGAA |
| LRP1-FP | CGAAGTGACCAACCCATGTG |
| LRP1-RP | CGTTGAAGCACTGCAGGTTA |
| STEAP3-FP | CTACAGCTTCTGCTTGCCG |
| STEAP3-RP | GTAGATCTCCATCCGCCAGA |
| CD163-FP | GCTGTGGTAACTTGCATCCTG |
| CD163-RP | GCAGTAGTGTTCCACCCATCA |
