# Supplementary figures and images for: Ferroptosis Activation Contributes to the Formation of Skin Lesions in Psoriasis Vulgaris
Source: Antioxidants (Basel). 2023 Jan 29;12(2):310. doi: 10.3390/antiox12020310 (PMC9952139; doi:10.3390/antiox12020310)

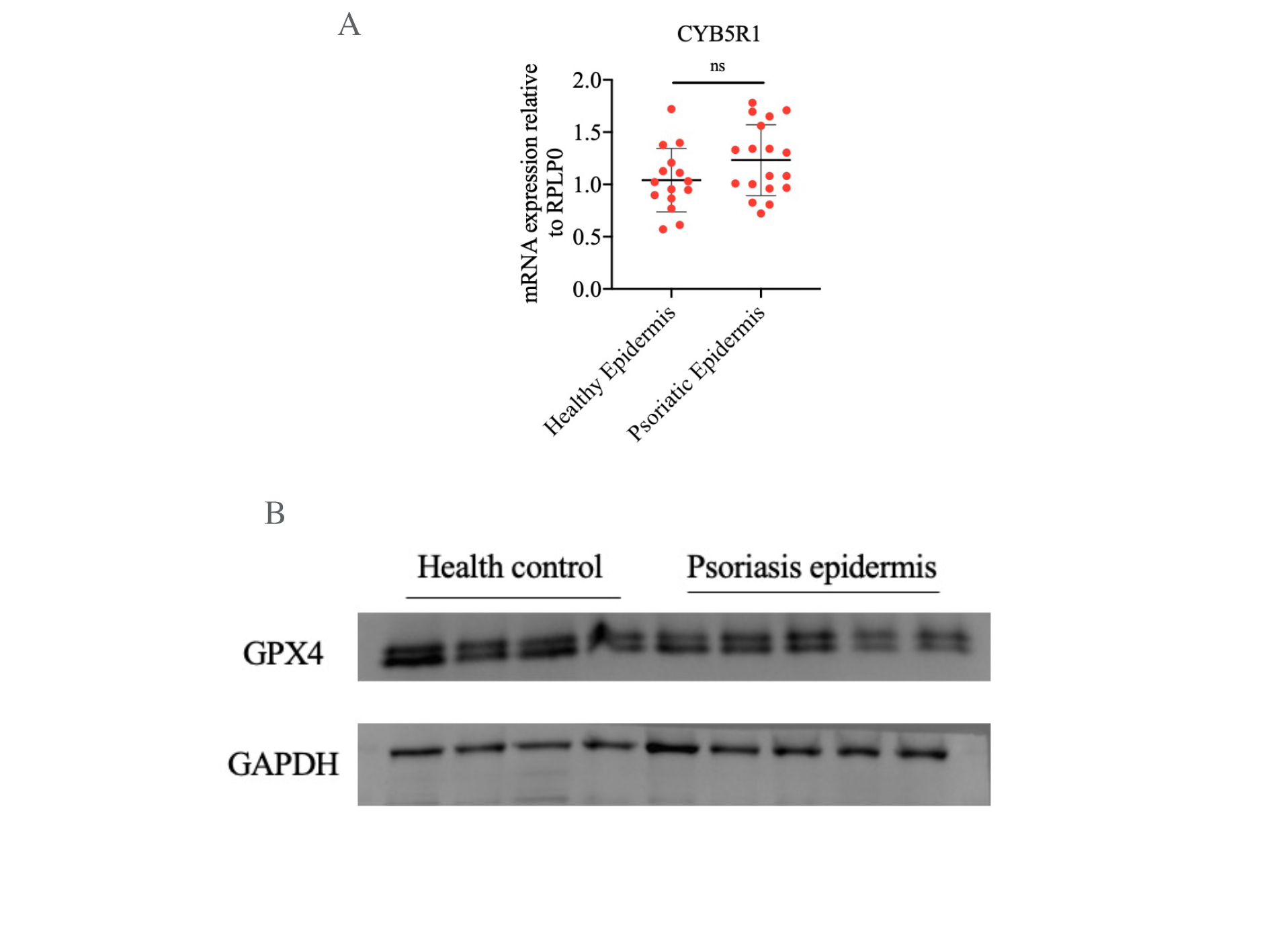

Supplement: Supplementary file 1 [file antioxidants-12-00310-s001.zip › antioxidants-2109138-supplementary/figure S2.jpg]
